# Prognostic value of pretreatment and recovery duration of cranial nerve palsy in nasopharyngeal carcinoma

**DOI:** 10.1186/1748-717X-7-149

**Published:** 2012-09-07

**Authors:** Hao-Yuan Mo, Rui Sun, Jian Sun, Qing Zhang, Wen-Jin Huang, Yan-Xian Li, Jing Yang, Hai-Qiang Mai

**Affiliations:** 1 State Key Laboratory of Oncology in South China, Sun Yat-sen University Cancer Center, No, 651 Dongfeng Road East, Guangzhou, 510060, People’s Republic of China; 2 Department of Nasopharyngeal Carcinoma, Sun Yat-sen University Cancer Center, 651 Dongfeng Road East, Guangzhou, 510060, People’s Republic of China; 3 Department of Clinical Trial Center, Sun Yat-sen University Cancer Center, No, 651 Dongfeng Road East, Guangzhou, 510060, People’s Republic of China; 4 Key Laboratory of Gene Engineering of the Ministry of Education, School of Life Sciences, Sun Yat-sen University, No, 135 Xingang Road West, Guangzhou, 510275, People’s Republic of China; 5 Department of Radiotherapy, Affiliated Cancer Hospital, Guangzhou Medical College, No, 78 Luhu Road, Guangzhou, 510095, People’s Republic of China

**Keywords:** Nasopharyngeal carcinoma, Cranial nerve involvement, Pretreatment duration, Recovery duration, Prognosis

## Abstract

**Background:**

The purpose of this study was to evaluate the prognostic value of cranial nerve (CN) palsy in nasopharyngeal carcinoma (NPC) patients.

**Methods:**

A retrospective analysis was performed on CN involvement using medical records of 178 consecutive patients with histologically diagnosed, non-disseminated NPC.

**Results:**

In 178 NPC patients with CN palsy, the 5-year survival rates were as follows: overall survival (OS), 61.0%; disease-specific survival (DSS), 69.6%; local relapse-free survival (LRFS), 75.2%; distant metastasis-free survival (DMFS), 73.4%; and disease-free survival (DFS), 55.3%. Significant differences were observed in the 5-year OS rates between patients with single and multiple CN palsy (69.8% vs. 54.3%; P = 0.033) and the OS rates between patients with different pretreatment durations (68.7% vs. 43.3%, P = 0.007). However, no significant differences were observed in OS, DSS, LRFS and DFS rates between patients with upper and lower CN palsy (P = 0.581, P = 0.792, P = 0.729 and P = 0.212, respectively). The results showed that recovery duration was an independent prognostic factor for OS (HR = 2.485; P < 0.001), DSS (HR = 2.065; P = 0.016), LRFS (HR = 3.051; P = 0.001) and DFS (HR = 2.440; P < 0.001).

**Conclusions:**

Recovery duration is an independent prognostic factor for NPC patients with CN palsy and is related to recurrence, which leads to poor survival. Recovery duration requires close surveillance and different treatment regimens.

## Background

Cranial nerve (CN) palsy in nasopharyngeal carcinoma (NPC) is the result of an adjacent extension of the primary tumor. CN palsy occurs in 8.0 to 12.4% of cases [1]. Cranial nerve involvement is considered an unfavourable prognostic factor for NPC patients, and those with CN palsy have been classified as T4 according to the staging system of NPC in the seventh edition of the American Joint Committee on Cancer (AJCC) [2]. The patients with extensive CN palsy had worse survival rates than those with only upper CN or lower CN involvement [3]. Moreover, the patients who recovered from CN palsy had better survival rates than those who did not. However, no recent research has indicated the prognostic value of the pretreatment or recovery durations for NPC patients with CN palsy.

The diagnosis of CN palsy mostly depends on clinical symptoms and a physical examination, and magnetic resonance imaging (MRI) has proven to be an important tool for defining CN involvement in NPC [4-6]. However, CN palsy frequently accompanies skull-base invasion in upper CN palsy and carotid sheath erosion in lower CN palsy. Skull-base invasion can approach from basicranial to intracranial or orbital sites, and carotid sheath erosion will extracranially involve lower CN palsy. Previous studies have focussed only on survival rates and imaging diagnoses in NPC patients with and without CN palsy, and those studies did not indicate the prognostic value of the pretreatment or recovery durations in NPC patients with CN palsy.

The National Comprehensive Cancer Network (NCCN) has recommended concurrent chemoradiotherapy plus adjuvant or induction chemotherapy as the standard treatment for patients with CN palsy. However, it is important to understand whether similar chemotherapy cycles and radiotherapy doses benefit NPC patients with CN palsy with different pretreatment and recovery durations. Hence, research on the prognostic value of the pretreatment and recovery durations of NPC patients with CN palsy was performed with a large sample size. Because the pretreatment program design is very important, in this study, we aimed to grade the pretreatment and recovery durations of NPC patients with CN palsy and to precisely judge patient prognosis. The results of this study may help to develop individualised treatment for NPC patients [7].

## Methods

### Patients

We reviewed the records of consecutive NPC patients referred to our center between January 1, 2002, and December 31, 2003. During the study period, there were 1,892 primary NPC patients who received radiation therapy in our institution. Of these patients, we identified 178 NPC patients with CN palsy (9.4%; 178/1892) who had completed radical treatment during the study period. All clinical records were reviewed by the authors. Of these patients, 145 patients were male, and 33 were female (a male-to-female ratio of 4.4:1). The patients had biopsy-proven NPC without distant metastases and were treated radically with radiation. The median age of the 145 male patients was 47.7 years (range, 18–74 years), and the median age of the 33 female patients was 45.3 years (range, 21–67 years).

The CN palsy diagnosis was mainly based on clinical symptoms and a physical examination. At histologic examination, 174 of the 178 patients (97.8%) were classified as having type II or III disease based on the World Health Organization criteria. Three patients (1.7%) were classified as having type I disease; the remaining patient (0.5%) had an adenocarcinoma. All patients underwent a pretreatment evaluation that included a complete patient history, physical and neurologic examinations, haematological and biochemical profiles, MR imaging of the neck and nasopharynx, chest radiography and abdominal ultrasonography. Medical records were retrospectively analysed, and all patients were staged according to the staging system of the seventh edition of the American Joint Committee on Cancer. The stage distribution for the patients was as follows: 10.1% of patients (18/178) belonged to clinical stage IVb with N3 classification; other patients belonged to clinical stage IVa with T4 classification, including 16.3% of patients (29/178) classified as N0; 51.7% (92/178) as N1; and 21.9% (39/178) as N2. Table 1 shows the characteristics of all patients.

**Table 1:** Patient characteristics (n = 178)

**Characteristics**	**Number**	**Percentage (%)**
**Age**	**Median: 46.5**	
	**(Range, 18–74)**	
Sex		
Male	145	81.5
Female	33	18.5
Biopsy (WHO)		
I	3	1.7
II + III	174	97.8
Adenocarcinoma	1	0.5
N stage*		
N0	29	16.3
N1	92	51.7
N2	39	21.9
N3	18	10.1
Radiotherapy		
2D-CRT	146	82.0
IMRT	24	13.5
3D-CRT	8	4.5
Chemotherapy		
No	44	24.7
Yes	134	75.3

§According to the AJCC seventh edition.

### Treatment

All patients were treated with radical radiotherapy. The details of the radiation therapy techniques used at the Cancer Center of Sun Yat-sen University have been described previously [8-10]. In brief, most patients (146/178; 82.0%) were treated with conventional techniques, 13.5% (24/178) received intensity-modulated radiation therapy (IMRT) and 4.5% (8/178) were administered 3-dimensional conformal radiation therapy (3D-CRT).

A total of 44 of 178 patients (24.7%) were treated with radiotherapy only. A total of 134 of 178 patients (75.3%) received chemotherapy, including various regimens of concurrent chemotherapy combined with either induction chemotherapy or adjuvant chemotherapy in conjunction with a platinum-based therapeutic clinical trial. Deviations from the guidelines were due to advanced age, heart disease, severe diabetes, or inadequate renal or hepatic function, which suggested intolerance to chemotherapy. When possible, salvage treatments, such as neck dissection and chemotherapy, were provided in the event of documented relapse or persistent disease.

### Follow-up and statistical analyses

The duration of patient follow-up was calculated from the first day of treatment to either the day of death or the day of the last examination. The patients were examined at least every 3 months during the first 2 years; thereafter, a follow-up examination was performed every 6 months for up to 5 years or until death. The time of last follow-up was March 2010, and the median follow-up period was 61.4 months (range, 2.5–86.6 months).

All events were measured from the date of treatment commencement. The following end points (time to the first defining event) were assessed: overall survival (OS); disease-specific survival (DSS); local relapse-free survival (LRFS); distant metastasis-free survival (DMFS); and disease-free survival (DFS). In calculating the disease-specific survival, a patient dying of their tumour was a point event; patients who survived, defaulted follow-up, or died of other disease were censored. Local recurrence was established by fibreoptic endoscopy and biopsy or MRI. Distant metastases were diagnosed on the basis of clinical symptoms, physical examination, and imaging methods, including chest radiography, bone scans, MRI and abdominal sonography.

All statistical analyses were performed using the Statistical Package for the Social Sciences (SPSS) 13.0 software (SPSS Inc., Chicago, IL). The actuarial rates were calculated by the Kaplan-Meier method, and differences were compared using the log-rank test. Multivariate analyses with the Cox proportional hazards model were used to calculate the hazard ratio (HR) and test independent significance by backward elimination of insignificant explanatory variables. Host factors (age and sex) were included as the covariates in all tests. The criterion for significance was set at 0.05, and P values were based on 2-sided test results.

## Results

### Incidence and Survival of CN Palsy

Of the 1892 patients with NPC, 178 (9.4%) had clinical evidence of CN palsy at presentation including 171 patients with unilateral palsy and 7 with bilateral palsy. The most common CN palsies among these patients involved VI, V2 and V3 (49.4%, 44.9% and 35.4%, respectively). In 178 T_4_ patients, the 5-year survival rates were as follows: overall survival (OS), 61.0%; disease-specific survival (DSS), 69.6%; local relapse-free survival (LRFS), 75.2%; distant metastasis-free survival (DMFS), 73.4%; and disease-free survival (DFS), 55.3%.

### Differences in the prognostic implications of single and multiple CN palsy

**Figure 1:**
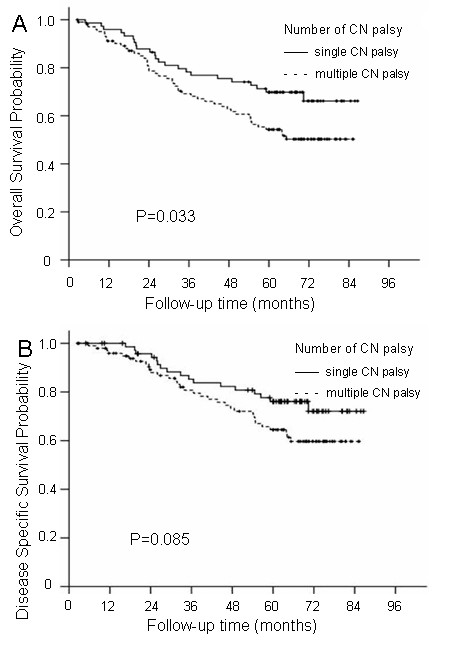
The overall survival and disease-specific survival curves for the different numbers of CN palsy groups: Group 1, patients with single CN palsy; Group 2, patients with multiple CN palsy (A and B).

Of the 178 patients, 74 patients (41.6%) had single CN palsy and 104 patients (58.4%) had multiple CN palsy. Significant differences were observed in the 5-year OS rates (69.8% vs. 54.3%; P = 0.033) between the two groups with single and multiple CN palsy, but no significant differences were observed for DSS (76.1% vs. 64.5%; P = 0.085), LRFS (73.2% vs. 76.8%; P = 0.783), DMFS (76.9% vs. 70.5%; P = 0.384) or DFS (59.0% vs. 52.7%; P = 0.317) rates between the two groups (Figure 1).

The following parameters, which could potentially influence patient prognosis, were included in the Cox proportional hazards model for multivariate analysis: age (>median age vs. ≤median age), sex, N classification, radiotherapy dose (>70 Gy vs. ≤70 Gy), chemotherapy, and CN palsy (with single and multiple involvement). The results indicated that the number of CN palsies was an independent prognostic factor for OS (HR = 1.765; P = 0.029), but not for DSS, LRFS, DMFS or DFS.

### Prognosis of upper and lower CN palsy

**Figure 2:**
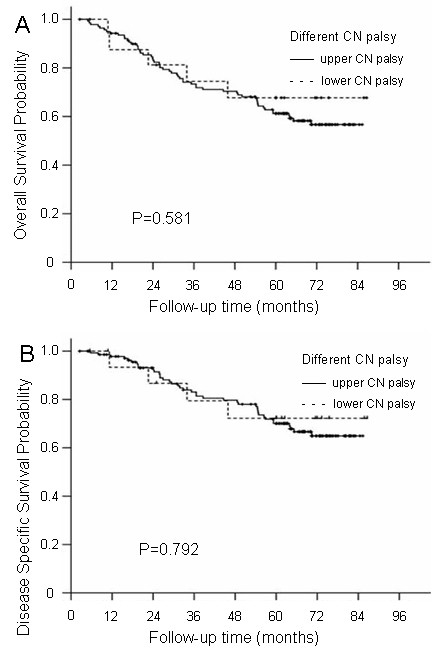
The overall survival and disease-specific survival curves for the different section groups: Group 1, patients with upper CN palsy; Group 2, patients with lower CN palsy (A and B).

Of the 178 patients, 140 patients (78.7%) had upper CN palsy and 17 patients (9.6%) had lower CN palsy. No significant differences were observed for OS (61.2% vs. 67.7%; P = 0.581), DSS (70.1% vs. 72.2%; P = 0.792), LRFS (75.4% vs. 73.9%; P = 0.729), DMFS (70.1% vs. 86.5%; P = 0.257) or DFS (52.7% vs. 73.9%; P = 0.212) rates between the two groups (Figure 2).

The following parameters, which could potentially influence patient prognosis, were included in the Cox proportional hazards model for multivariate analysis: age (>median age vs. ≤median age), sex, N classification, radiotherapy dose (>70 Gy vs. ≤70 Gy), chemotherapy, and CN palsy (with upper and lower involvement). The results indicated that the location of CN palsy was not an independent prognostic factor for OS, DSS, LRFS, DMFS or DFS.

### Prognostic value of different pretreatment durations

**Figure 3:**
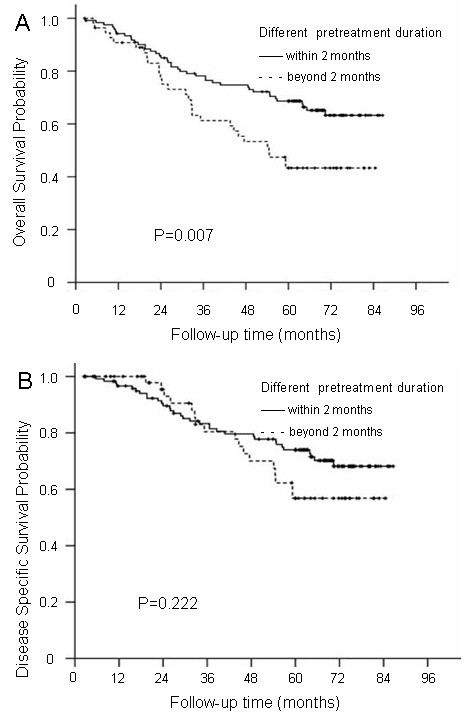
The overall survival and disease-specific survival curves for the different pretreatment duration groups: Group 1, patients with CN palsy with a pretreatment duration within 2 months; Group 2, patients with a pretreatment duration beyond 2 months (A and B).

Because the patients with CN palsy had different duration times before clinical diagnosis, it is essential to determine the prognostic value of the pretreatment duration. The patients with CN palsy in this series were classified into two groups: Group 1 included patients with CN palsy with a pretreatment duration within 2 months, and Group 2 had a pretreatment duration longer than 2 months. A significant difference was observed for OS (68.7% vs. 43.3%; P = 0.007) between Groups 1 and 2, but no significant differences were observed for DSS (74.0% vs. 56.8%; P = 0.222), LRFS (78.9% vs. 66.8%; P = 0.068), DMFS (72.4% vs. 74.7%; P = 0.532) or DFS (57.5% vs. 49.7%; P = 0.369) rates between the two groups (Figure 3).

A multivariate analysis was performed to adjust for various prognostic factors in Group 1 and 2 patients with different pretreatment durations of CN palsy. The following parameters were included in the Cox proportional hazards model by backward elimination of insignificant explanatory variables: age (>median age vs. ≤median age), sex, N classification, radiotherapy dose (>70 Gy vs. ≤70 Gy), chemotherapy, and pretreatment duration (Groups 1 and 2). The results indicated that pretreatment duration was an independent prognostic factor for OS (HR = 1.926; P = 0.009) but not for DSS, LRFS, DMFS or DFS.

### Further prognostic analysis of patients with different recovery durations

**Figure 4:**
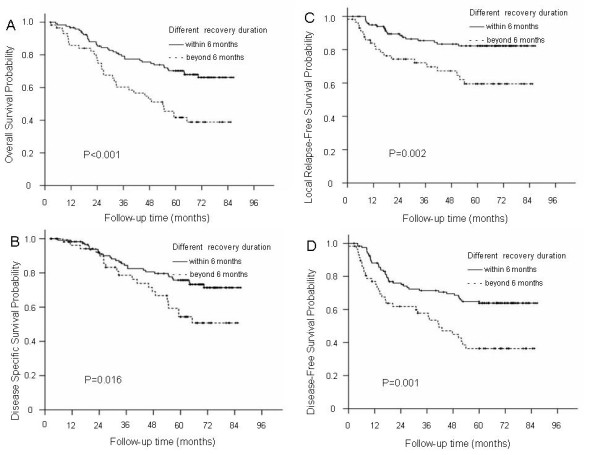
The overall survival, disease-specific survival, local relapse-free survival and disease-free survival curves for the different recovery duration groups: Group 1, recovery duration of patients with CN palsy within 6 months; Group 2, recovery duration beyond 6 months (A/B/C/D).

Because most patients recover from CN palsy within six months, all patients with CN palsy in this series were classified into two groups: Group 1 included patients with a recovery duration within 6 months, and Group 2 included patients with a recovery duration beyond 6 months. Significant differences were observed for 5-year OS (69.1% vs. 41.7%; P < 0.001), DSS (75.8% vs. 54.4%; P = 0.016), LRFS (82.3% vs. 59.5%; P = 0.002) and DFS (63.9% vs. 36.4%; P = 0.001) rates between Groups 1 and 2, but no significant difference was observed for DMFS (75.6% vs. 66.8%; P = 0.405) rates between Groups 1 and 2 (Figure 4).

A multivariate analysis was performed to adjust for various prognostic factors in Group 1 and 2 patients with different recovery durations of CN palsy. The following parameters were included in the Cox proportional hazards model by backward elimination of insignificant explanatory variables: age (>median age vs. ≤median age), sex, N classification, radiotherapy dose (>70 Gy vs. ≤70 Gy), chemotherapy, and recovery duration (Groups 1 and 2). The results indicated that recovery duration was an independent prognostic factor for OS (HR = 2.485; P < 0.001), DSS (HR = 2.065; P = 0.016), LRFS (HR = 3.051; P = 0.001) and DFS (HR = 2.440; P < 0.001) but not for DMFS.

## Discussion

In this study, the incidence of cranial nerve palsy (9.4%) was similar to that found in previous research (approximately 8.0 to 12.4%) [1]. The AJCC has recommended that CN involvement be assessed through neurological evaluation rather than cross-sectional imaging [2]. Because CN involvement is considered a poor prognostic indicator in NPC patients, those with CN involvement are staged as T4. CN palsy frequently accompanied skull-base invasion in upper CN palsy and carotid sheath erosion in lower CN palsy. Moreover, skull-base invasion frequently approaches from basicranial to intracranial or orbital sites and involves upper CNs. In addition, the CN V and VI are located in the sites most prone to tumor invasion and extend a long distance; their involvement can often be observed.

The results of this study showed that NPC patients with multiple CN palsy have poorer OS than those with single CN palsy, but the difference in disease-specific survival rates was not significant. Patient prognosis is likely related to the extent and volume of the tumor, and the incorporation of the primary tumor volume may lead to a further refinement of treatment outcome [3,11]. NPC patients with multiple CN palsy may require more cycles of induction or adjuvant chemotherapy in addition to the base of concomitant chemotherapy and radiation [12,13]. However, the results of our study revealed that the DSS was not significant for different groups with single and multiple CN palsy, which may account for the observation that different numbers of CN palsy were not the crucial cause of death. Moreover, CN palsy with upper or lower involvement was not found to be a significant factor influencing OS, DSS, LRFS or DFS. This result was likely related to anatomy. CN palsy often accompanies basicranial, intracranial or orbital invasion in upper CN palsy and carotid sheath erosion in lower CN palsy. Upper CN involvement was associated with recurrence from the primary invasion; in contrast, lower CN involvement accompanied lymph node erosion, which could increase metastasis, exhibiting the hazard criteria consistent with the staging classification system [14]. Advanced T and N stages had similar prognoses.

In 2010, the NCCN recommended that the treatment regimen for NPC patients with CN involvement should consist of concurrent chemoradiotherapy plus adjuvant or induction chemotherapy as the standard treatment. Despite the fact that the pretreatment duration was associated with significantly poorer OS in this study, the difference in DSS was not significant between the groups with different pretreatment durations. Moreover, no other study has assessed the relationship between pretreatment duration and prognosis. Further studies are required to determine whether more regimens and cycles of chemotherapy would add a benefit to radiotherapy for patients with different pretreatment durations. These findings may have implications for further improvements in treating advanced-stage tumors with different pretreatment durations.

In our study, recovery duration was an independent prognostic factor for 5-year LRFS in patients with NPC. In a previous study, CN recovery correlated significantly with the pretreatment duration of CN palsy, the time course of clinical tumor regression, and neurologic symptom improvement during radiation [15], which recommended a need for continuous and close neurologic surveillance. In addition, patients with NPC had a higher rate of radiation-induced CN palsy, with a median latent period of 7.6 years (range, 0.3-34 years) [16]. It is essential to examine CN involvement before treatment and to estimate the cause from clinical data; in particular, MRI has been shown to be a valuable tool for detecting and defining the extent of CN involvement in NPC [17]. The results of our study showed that patients whose recoveries from CN palsy required longer than six months had poorer OS, DSS, LRFS and DFS. We hypothesize that recovery duration is related to recurrence, which leads to poor survival. Thus, patients with longer recovery durations require follow-up after six months to detect recurrence and sequelae that influence patient prognosis.

## Conclusions

In conclusion, recovery duration is an independent prognostic factor for NPC with CN palsy and is related to recurrence that leads to poor survival. Moreover, recovery duration requires close surveillance and different treatment regimens.

## Abbreviations

AJCC: American joint committee on cancer; NCCN: National comprehensive cancer network; NPC: Nasopharyngeal carcinoma; CN: Cranial nerve; OS: Overall survival; DSS: Disease-specific survival; LRFS: Local relapse-free survival; DMFS: Distant metastasis-free survival; DFS: Disease-free survival.

## Misc

Hao-Yuan Mo and Rui Sun contributed equally to this work.

## Competing interests

The authors declare that they have no competing interests.

## Authors’ contributions

Guarantors of integrity of the entire study: H-YM, RS, H-QM; study concepts/study design, data acquisition or data analysis/interpretation: all authors; manuscript drafting or revision for important intellectual content: all authors; manuscript final version approval: all authors; literature review: R. Sun, H-Q Mai; clinical studies: RS, W-JH, Y-XL, JY; statistical analyses: RS, JS, QZ, W-JH; and manuscript editing: RS, JS, QZ.

## Authors’ information

H-Y Mo **(first author)**

R. Sun **(co-first author)**
